# Both Comprehensive and Brief Self-Administered Diet History Questionnaires Satisfactorily Rank Nutrient Intakes in Japanese Adults

**DOI:** 10.2188/jea.JE20110075

**Published:** 2012-03-05

**Authors:** Satomi Kobayashi, Satoru Honda, Kentaro Murakami, Satoshi Sasaki, Hitomi Okubo, Naoko Hirota, Akiko Notsu, Mitsuru Fukui, Chigusa Date

**Affiliations:** 1 Department of Social and Preventive Epidemiology, Graduate School of Medicine, The University of Tokyo, Tokyo, Japan; 2 Department of Social and Preventive Epidemiology, School of Public Health, The University of Tokyo, Tokyo, Japan; 3 Research Fellow of the Japan Society for the Promotion of Science; 4 Department of Health and Nutritional Science, Faculty of Human Health Science, Matsumoto University, Nagano, Japan; 5 Department of Food Science and Nutrition, Tottori College, Tottori, Japan; 6 Department of Statistics, Osaka City University Medical School, Osaka, Japan; 7 Department of Food Science and Nutrition, School of Human Science and Environment, University of Hyogo, Hyogo, Japan

**Keywords:** diet history questionnaire, nutrient intake, relative validity, Japanese

## Abstract

**Background:**

A comprehensive self-administered diet history questionnaire (DHQ: 150-item semi-quantitative questionnaire) and a brief self-administered DHQ (BDHQ: 58-item fixed-portion–type questionnaire) were developed for assessing Japanese diets. We compared the relative validity of nutrient intake derived from DHQ with that from the BDHQ, using semi-weighed 16-day dietary records (DRs) as reference.

**Methods:**

Ninety-two Japanese women aged 31 to 69 years and 92 Japanese men aged 32 to 76 years completed a 4-nonconsecutive-day DR, a DHQ, and a BDHQ 4 times each (once per season) in 3 areas of Japan (Osaka, Nagano, and Tottori).

**Results:**

No significant differences were seen in estimates of energy-adjusted intakes of 42 selected nutrients (based on the residual method) between the 16-day DRs and the first DHQ (DHQ1) or between the DR and the first BDHQ (BDHQ1) for 18 (43%) and 14 (33%) nutrients, respectively, among women and for 4 (10%) and 21 (50%) nutrients among men. The median (interquartile range) Pearson correlation coefficients with the DR for energy-adjusted intakes of the 42 nutrients were 0.57 (0.50 to 0.64) for the DHQ1 and 0.54 (0.45 to 0.61) for the BDHQ1 in women; in men, the respective values were 0.50 (0.42 to 0.59) and 0.56 (0.41 to 0.63). Similar results were observed for the means of the 4 DHQs and BDHQs.

**Conclusions:**

The DHQ and BDHQ had satisfactory ranking ability for the energy-adjusted intakes of many nutrients among the present Japanese population, although these instruments were satisfactory in estimating mean values for only a small number of nutrients.

## INTRODUCTION

Dietary questionnaires are useful tools for assessing long-term dietary habits.^1^ However, because food culture and dietary habits vary by country, these questionnaires need to be developed specifically for each country.^2^ To assess Japanese diets, Sasaki et al developed a comprehensive self-administered diet history questionnaire (DHQ) and a brief self-administered DHQ (BDHQ), which use both the food frequency and diet history methodologies.^3^^,^^4^ The DHQ yields information on the dietary intake of 150 food and beverage items but requires about 45 to 60 minutes to answer, whereas the BDHQ provides information on only 58 items but requires only about 15 to 20 minutes to complete.

Because dietary questionnaires do not necessarily estimate true food intake, their validity should be evaluated. Several US studies have compared the validity of short and long versions of questionnaires,^5^^–^^7^ but no such studies have been reported in other countries. We previously examined the validity of the DHQ and BDHQ in terms of food group intake and found that they had similar and satisfactory validity for a wide range of food groups among adult Japanese women and men in terms of their ability to both estimate median values and rank individuals in a population.^4^ However, the validity of these questionnaires with regard to nutrient intake has not been compared. Because a finding of satisfactory validity for food groups does not reflect satisfactory validity for nutrients, we must examine the validity of nutrient intakes for the DHQ and BDHQ despite the existence of a validation study for food group intakes.

Here, we compared the relative validity of energy and nutrient intakes derived from the DHQ and BDHQ among healthy women and men in Japan against 16-day semi-weighed dietary records (DRs).

## METHODS

### Study design

Details of the study design, participant characteristics, and dietary assessment methods have been reported elsewhere.^4^^,^^8^ Briefly, the study was conducted in 3 areas of Japan, namely, Osaka (urban), Nagano (rural inland), and Tottori (rural coastal). In each area, we recruited apparently healthy women aged 30 to 69 years who were willing to participate with a cohabitating husband, such that each of the 10-year age class strata had 8 women, regardless of the age of the husbands. Thus, a total of 96 women and 96 men were invited. Before the study, the study purpose and protocol were explained at group orientations. Written informed consent was obtained from each participant. The study did not undergo ethical approval because it was conducted before the advent of ethical guidelines for epidemiology research in Japan. However, the use of the data of this study was approved by the Ethics Committee at the University of Tokyo Faculty of Medicine (No. 3421). Ultimately, 92 women aged 31 to 69 years and 92 men aged 32 to 76 years who completed the protocol were included in the present analysis.

Between November 2002 and September 2003, the participants completed four 4-nonconsecutive-day semi-weighed DRs, 1 in each season, at intervals of approximately 3 months, ie, November and December 2002 (autumn), February 2003 (winter), May 2003 (spring), and August and September 2003 (summer) (Figure). Each of the 4 recording days consisted of 1 weekend day and 3 weekdays within about 2 weeks. During the orientation session, registered dietitians gave the participants both written and verbal instructions on how to keep the DR, provided recording sheets and a digital scale, and asked the participants to record and weigh all foods and beverages consumed on the recording day. All collected records were checked by trained registered dietitians in the respective local center and then again in the study center. A total of 1299 food and beverage items appeared in the dietary records.

**Figure.**
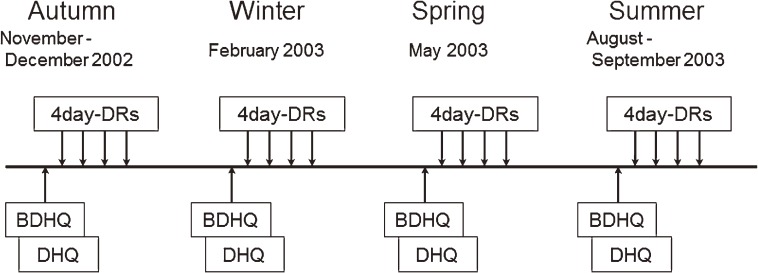
Schedule for the present validation study. Abbreviations: DR = semi-weighed dietary records; DHQ = self-administered diet history questionnaire; BDHQ = brief self-administered diet history questionnaire.

The participants also answered the DHQ and BDHQ 4 times, once in each season, at intervals of approximately 3 months from November 2002 to September 2003. In each season, the DHQ and BDHQ were answered approximately 2 days before the start of the dietary recording period, in the order of BDHQ before DHQ (Figure). Responses to the DHQ and BDHQ were checked at least twice for completeness by dietitians. When missing answers or logical errors were identified, the participants were asked to complete the questions again.

The DHQ is a 16-page semi-quantitative questionnaire that asks about the consumption frequency and portion size of selected foods to estimate the dietary intake of 150 food and beverage items during the preceding month.^3^^,^^4^^,^^8^^–^^11^ The DHQ consists of 7 sections: (1) general dietary behavior, (2) usual cooking methods, (3) consumption frequency and amount of alcoholic beverages, (4) consumption frequency and semi-quantitative portion size of selected food and nonalcoholic beverage items, (5) dietary supplements, (6) consumption frequency and semi-quantitative portion size of staple foods, soup for noodles, and miso (fermented soybean paste) soup, and (7) open-ended items for foods consumed more than once a week but not appearing in the DHQ.

The BDHQ is a 4-page fixed-portion questionnaire that asks about the consumption frequency of selected foods, but not about portion size, to estimate the dietary intake of 58 food and beverage items during the preceding month.^4^ The BDHQ consists of 5 sections: (1) intake frequency of food and nonalcoholic beverage items, (2) daily intake of rice and miso soup, (3) frequency of drinking and amount per drink for alcoholic beverages, (4) usual cooking methods, and (5) general dietary behavior.

Food and beverage items contained in the DHQ and BDHQ were selected from foods commonly consumed in Japan, mainly from a food list used in the National Health and Nutrition Survey of Japan,^12^ while standard portion sizes and the sizes of bowls for rice and cups for miso soup were derived from several recipe books for Japanese dishes.^3^^,^^4^ Because the use of dietary supplements is uncommon in Japan (8% of the general population),^12^ we did not considered information on dietary supplements that was derived from the DHQ.

### Statistical analysis

All statistical analyses were done using SAS statistical software, version 9.2 (SAS Institute Inc., Cary, NC, USA) and were performed separately for women and men. Crude values for the intake of energy and 42 selected nutrients were estimated based on the intake of food items obtained with the DR or respective questionnaire and the corresponding food composition list in the *Standard Tables of Food Composition in Japan*.^13^ Because intakes of most nutrients were positively correlated with energy intake, energy-adjusted values were also calculated by the residual method using a regression model, while the density method was used to compute the amount of each nutrient consumed daily, as a percentage from daily energy intake for energy-containing nutrients or per 10 MJ of daily energy intake for non–energy-containing nutrients.^14^ All statistical analyses were performed on log-transformed values to account for non-normality. Relative validity of the DHQ and BDHQ were assessed in terms of their ability to estimate representative values by comparing mean values and ranking ability, using Pearson correlation coefficients. We compared nutrient intakes derived from the first DHQ (DHQ1) and the first BDHQ (BDHQ1) with those from the four 4-day DRs. Although the reference period differed between DHQ1 or BDHQ1 and the four 4-day DRs (1 conducted in each season), our purpose here was to examine whether a single DHQ or BDHQ for dietary habits during the previous month could represent habitual dietary intake over a longer period. Additionally, we also examined the mean of the 4 DHQs and BDHQs (mDHQ and mBDHQ) using the same method to compare annual intake from the questionnaires with that of the DR.

Energy and nutrient intakes in the crude and energy-adjusted models for DR, DHQ1, BDHQ1, mDHQ, and mBDHQ are presented as means. Statistically significant differences between the DR and each of the questionnaires were determined with the paired *t*-test using 1-sided values. A *P* value less than 0.05 was considered to indicate a significant difference. Pearson correlation coefficients between the DR and DHQ1, BDHQ1, mDHQ, and mBDHQ were then calculated. Additionally, because a 16-day DR may be insufficient to account for intra-individual variation, we also calculated deattenuated correlation coefficients using intraindividual and interindividual variances.^1^ Correlation coefficients obtained from the DHQ and BDHQ were compared using the Meng-Rosenthal-Rubin method to compare overlapping correlation coefficients,^15^ with tested correlation coefficient pairs considered statistically different when z was greater than 1.96, with a significance level of 5%.

## RESULTS

Selected characteristics of the participants are shown in Table 1. The means of energy and nutrient intakes derived from the DR, DHQ, and BDHQ are shown in Table 2 for women and Table 3 for men. Energy intakes estimated by BDHQ1 and mBDHQ for women and by DHQ1, BDHQ1, and mBDHQ for men were significantly lower than those of the DR. Regarding crude intakes of the 42 nutrients, no significant differences between the DR and DHQ1 and between the DR and BDHQ1 were observed for 28 (67%) and 18 (43%) nutrients, respectively, for women and 11 (26%) and 27 (64%) nutrients for men (data not shown). Regarding nutrients that were energy-adjusted by the residual method, no significant differences between the DR and DHQ1 and between the DR and BDHQ1 were observed for 18 (43%) and 14 (33%) nutrients, respectively, for women and 4 (10%) and 21 (50%) nutrients for men. Regarding nutrients that were energy-adjusted by the density method, no significant differences between the DR and DHQ1 and between the DR and BDHQ1 were observed for 21 (50%) and 8 (19%) nutrients, respectively, for women and 13 (31%) and 16 (38%) nutrients for men. For mDHQ and mBDHQ, the numbers of nutrients that did not significantly differ between the DR and mDHQ and between the DR and mBDHQ were similar to those that did not significantly differ between the DHQ1 and BDHQ1. For women, the respective numbers of nutrients for the crude, residual, and density methods were 22 (52%), 15 (36%), and 17 (40%) for the mDHQ and 19 (45%), 12 (29%), and 7 (17%) for the mBDHQ. The respective values for men were 14 (33%), 11 (26%), and 15 (36%) for the mDHQ and 24 (57%), 15 (36%), and 11 (26%) for the mBDHQ.

**Table 1.** Selected characteristics of the participants (92 women and 92 men)

	Women		Men	
	
	Mean	SD	Mean	SD
Age (years)	49.6	11.4	52.8	12.1
Body height (cm)	155.6	5.8	168.0	6.7
Body weight (kg)	53.4	7.1	66.2	11.2
BMI (kg/m^2^)	22.1	2.6	23.3	3.1

Abbreviation: SD = standard deviation.

**Table 2.** Comparison of mean daily energy intake and crude and energy-adjusted^a^ nutrient intakes estimated by the 16-day DR, DHQ1, BDHQ1, mDHQ, and mBDHQ among 92 women

	Crude and energy-adjusted by the residual method^b^						Energy-adjusted by the density method					
	
	Unit	DR	DHQ1	BDHQ1	mDHQ	mBDHQ	Unit	DR	DHQ1	BDHQ1	mDHQ	mBDHQ
Energy	kJ/d	7722	7858	7167**	7741	7008***		—	—	—	—	—
Protein	g/d	69.5	65.8***	67.2*	64.9***	65.8***	% energy	15.1	14.0***	15.7**	14.0***	15.7**
Fat	g/d	56.1	57.3	48.0***	56.9	47.7***	% energy	27.3	27.5	25.2***	27.7	25.6***
Saturated fat	g/d	15.8	15.5	13.0***	15.8	13.1***	% energy	7.71	7.43	6.84***	7.66	7.05***
Monounsaturated fat	g/d	19.5	19.9	16.6***	19.8	16.5***	% energy	9.50	9.51	8.74***	9.63	8.87***
Polyunsaturated fat	g/d	12.8	13.4*	11.5***	13.3*	11.4***	% energy	6.23	6.42	6.02	6.45	6.12
*n*-6 polyunsaturated fat	g/d	10.3	10.8*	9.0***	10.7**	8.9***	% energy	5.00	5.17	4.71**	5.22*	4.78*
*n*-3 polyunsaturated fat	g/d	2.45	2.64*	2.45	2.58*	2.46	% energy	1.20	1.26	1.28*	1.25	1.31***
Marine-origin *n-3* polyunsaturated fat^c^	g/d	0.89	0.79*	0.93	0.78***	0.95	% energy	0.44	0.38**	0.48*	0.38***	0.51***
Eicosapentaenoic acid	g/d	0.30	0.28	0.32	0.27**	0.33*	% energy	0.15	0.13	0.17*	0.13**	0.18***
Docosahexaenoic acid	g/d	0.51	0.45*	0.53	0.44***	0.55	% energy	0.25	0.22**	0.28	0.22***	0.29**
α-linolenic acid	g/d	1.43	1.73***	1.41	1.69***	1.39	% energy	0.70	0.82***	0.74	0.82***	0.74**
Cholesterol	mg/d	334	303**	364**	306**	362**	mg/10 MJ	432	386**	508***	395**	516***
Carbohydrate	g/d	254	260**	240***	256	232***	% energy	55.1	55.5	56.0	55.3	55.5
Total dietary fiber	g/d	14.5	13.8*	13.5**	12.8***	12.0***	g/10 MJ	18.8	17.6**	18.9	16.6***	17.2***
Soluble dietary fiber	g/d	3.20	3.31	3.45**	3.11	3.01**	g/10 MJ	4.15	4.21	4.80***	4.01	4.28
Insoluble dietary fiber	g/d	10.5	10.0**	9.7***	9.2***	8.6***	g/10 MJ	13.7	12.7***	13.5	11.9***	12.3***
Alcohol	g/d	1.73	1.20***	1.05***	1.35**	1.24***	% energy	0.78	0.62*	0.57**	0.65**	0.65
Retinol	µg/d	256	241	339***	283	363***	µg/10 MJ	331	307	473***	366	518***
Vitamin A (retinol equivalent)^d^	µg/d	572	563	713***	580	699***	µg/10 MJ	741	717	995***	749	997***
α-carotene	µg/d	371	343	435*	319**	399	µg/10 MJ	480	437	607**	412**	569**
β-carotene	µg/d	2907	2814	3621***	2783	3331***	µg/10 MJ	3764	3581	5052***	3595	4754***
β-carotene equivalent^e^	µg/d	3344	3278	4102***	3133	3732**	µg/10 MJ	4330	4172	5724***	4047	5325***
Cryptoxanthin	µg/d	274	397***	338*	281	308	µg/10 MJ	355	506***	472**	363	439**
α-tocopherol	mg/d	7.40	7.70	7.33	7.77**	6.99***	mg/10 MJ	9.57	9.79	10.20**	10.01*	9.95*
Vitamin K	µg/d	228	259**	301***	252**	286***	µg/10 MJ	295	330**	420***	325**	408***
Thiamin	mg/d	0.90	0.84***	0.81***	0.82***	0.76***	mg/10 MJ	1.17	1.05***	1.12*	1.05***	1.07***
Riboflavin	mg/d	1.33	1.37	1.35	1.35	1.30	mg/10 MJ	1.71	1.73	1.87***	1.74	1.85***
Niacin	mg/d	16.5	15.9	16.4	15.3***	15.9	mg/10 MJ	21.4	20.2**	22.8**	19.8***	22.7**
Vitamin B_6_	mg/d	1.21	1.14***	1.31***	1.10***	1.22	mg/10 MJ	1.57	1.44***	1.80***	1.41***	1.72***
Vitamin B_12_	µg/d	7.35	7.07	8.59***	7.03	9.03***	µg/10 MJ	9.55	9.03	12.0***	9.10	12.9***
Folate	µg/d	349	308***	378**	295***	345	µg/10 MJ	452	392***	527***	381***	492***
Pantothenic acid	mg/d	6.01	6.11	6.54***	5.98	6.24**	mg/10 MJ	7.78	7.76	9.10***	7.71	8.88***
Vitamin C	mg/d	107	116	144***	101*	121***	mg/10 MJ	139	147	201***	130*	173***
Sodium	mg/d	4109	4215	3912**	4033	3860***	mg/10 MJ	5320	5365	5459	5210	5508*
Potassium	mg/d	2631	2472**	2796**	2357***	2577	mg/10 MJ	3408	3145***	3902***	3044***	3678***
Calcium	mg/d	563	551	579	541	558	mg/10 MJ	729	701	808***	699	797***
Magnesium	mg/d	270	255***	264	250***	251***	mg/10 MJ	350	325***	368**	323***	358
Phosphorus	mg/d	1068	1033*	1058	1017***	1031**	mg/10 MJ	1383	1315**	1476***	1314***	1471***
Iron	mg/d	8.14	7.12***	7.94	6.92***	7.57***	mg/10 MJ	10.5	9.0***	11.1*	8.9***	10.8
Zinc	mg/d	8.07	7.86*	8.05	7.79***	7.81**	mg/10 MJ	10.4	10.0***	11.2***	10.1***	11.1***
Copper	mg/d	1.18	1.13**	1.20	1.11***	1.14**	mg/10 MJ	1.52	1.44***	1.65***	1.43***	1.61***
Manganese	mg/d	3.56	4.06***	3.38*	3.84***	3.30***	mg/10 MJ	4.61	5.17***	4.72	4.97***	4.71

Abbreviations: DR = semi-weighed dietary records; DHQ1 = first self-administered comprehensive diet history questionnaire; BDHQ1 = first brief DHQ; mDHQ = mean of 4 DHQs; mBDHQ = mean of 4 BDHQs.

All variables were log-transformed before analysis. Means were back-transformed.

^a^Energy adjustment was performed according to the residual method and density method.

^b^Mean values obtained by the crude and residual methods were the same. The results of the paired *t*-test for the residual method are shown.

^c^Sum of eicosapentaenoic acid, docosapentaenoic acid, and docosahexaenoic acid.

^d^Sum of retinol, β-carotene/12, α-carotene/24, and cryptoxanthin/24.

^e^Sum of β-carotene, α-carotene/2, and cryptoxanthin/2.

Significant difference from the DR: *P < 0.05, **P < 0.01, ***P < 0.001 (paired *t*-test).

**Table 3.** Comparison of mean daily energy intake and crude and energy-adjusted^a^ nutrient intakes estimated by the 16-day DR, DHQ1, BDHQ1, mDHQ, and mBDHQ among 92 men

	Crude and energy-adjusted by the residual method^b^						Energy-adjusted by the density method					
	
	Unit	DR	DHQ1	BDHQ1	mDHQ	mBDHQ	Unit	DR	DHQ1	BDHQ1	mDHQ	mBDHQ
Energy	kJ/d	9804	9318*	8923**	9579	9046***		—	—	—	—	—
Protein	g/d	83.1	71.7***	74.9***	74.1***	77.7***	% energy	14.2	12.9***	14.1	12.9***	14.4
Fat	g/d	64.1	57.4***	51.7***	61.8*	55.0***	% energy	24.6	23.2*	21.8***	24.3	22.9***
Saturated fat	g/d	17.4	14.8***	13.4***	16.2***	14.4***	% energy	6.68	5.96***	5.64***	6.38**	5.99***
Monounsaturated fat	g/d	22.7	20.4***	18.2***	22.2	19.4***	% energy	8.72	8.26*	7.69***	8.74	8.08***
Polyunsaturated fat	g/d	14.8	13.9**	12.9***	14.8	13.6***	% energy	5.68	5.60	5.44	5.80	5.66
*n*-6 polyunsaturated fat	g/d	11.8	11.2*	10.1***	11.9	10.6***	% energy	4.52	4.52	4.25**	4.69	4.42
*n*-3 polyunsaturated fat	g/d	2.95	2.75*	2.78	2.89	2.94	% energy	1.13	1.11	1.17	1.14	1.22**
Marine-origin *n-3* polyunsaturated fat^c^	g/d	1.14	0.90***	1.05	0.93***	1.14	% energy	0.44	0.36***	0.44	0.37***	0.47
Eicosapentaenoic acid	g/d	0.39	0.32***	0.37	0.32***	0.40	% energy	0.15	0.12**	0.15	0.12***	0.16
Docosahexaenoic acid	g/d	0.66	0.52***	0.61	0.54***	0.66	% energy	0.25	0.21***	0.25	0.21***	0.27
α-linolenic acid	g/d	1.62	1.73*	1.59	1.84***	1.65	% energy	0.62	0.69***	0.67*	0.72***	0.69***
Cholesterol	mg/d	392	336***	392	365**	431***	mg/10 MJ	400	361**	440**	382	476***
Carbohydrate	g/d	314	303**	291***	306**	290***	% energy	53.7	54.4	54.6	53.5	53.7
Total dietary fiber	g/d	15.2	12.7***	13.9***	12.5***	13.0***	g/10 MJ	15.5	13.7***	15.5	13.0***	14.3***
Soluble dietary fiber	g/d	3.27	2.95***	3.41	2.97***	3.17	g/10 MJ	3.35	3.18	3.83***	3.10***	3.51*
Insoluble dietary fiber	g/d	11.1	9.3***	10.1***	9.1***	9.4***	g/10 MJ	11.4	10.0***	11.3	9.5***	10.4***
Alcohol	g/d	9.73	9.76	8.89	11.06	9.86	% energy	3.40	3.74	3.53	4.02**	3.80
Retinol	µg/d	287	224*	353*	295	418***	µg/10 MJ	293	241	395**	308	462***
Vitamin A (retinol equivalent)^d^	µg/d	620	485***	718*	550*	761***	µg/10 MJ	633	521**	805***	574	841***
α-carotene	µg/d	410	205***	340	228***	381	µg/10 MJ	418	221***	384	238***	422
β-carotene	µg/d	3043	2130***	3383	2308***	3335*	µg/10 MJ	3104	2287***	3792***	2410***	3686***
β-carotene equivalent^e^	µg/d	3453	2472***	3794	2585***	3711	µg/10 MJ	3522	2654***	4253**	2699***	4103***
Cryptoxanthin	µg/d	226	294*	275	225	273*	µg/10 MJ	232	317**	309*	235	302**
α-tocopherol	mg/d	8.21	7.67**	7.67**	8.24	7.87*	mg/10 MJ	8.37	8.22	8.57	8.59	8.68**
Vitamin K	µg/d	236	233	302***	234	299***	µg/10 MJ	241	250	339***	244	331***
Thiamin	mg/d	1.07	0.89***	0.87***	0.92***	0.86***	mg/10 MJ	1.09	0.95***	0.96***	0.95***	0.94***
Riboflavin	mg/d	1.48	1.36***	1.43	1.43*	1.46	mg/10 MJ	1.51	1.45	1.59*	1.49	1.61***
Niacin	mg/d	21.0	18.4***	19.5**	18.9***	19.8**	mg/10 MJ	21.4	19.7 **	21.8	19.7***	21.9
Vitamin B_6_	mg/d	1.47	1.30***	1.50	1.30***	1.47	mg/10 MJ	1.49	1.37***	1.66***	1.34***	1.61***
Vitamin B_12_	µg/d	9.01	8.05*	9.73	8.37	10.89***	µg/10 MJ	9.25	8.64	10.9**	8.75	12.1***
Folate	µg/d	385	297***	399	303***	386	µg/10 MJ	392	319***	448***	316***	426***
Pantothenic acid	mg/d	6.98	6.34***	7.06	6.48***	7.14*	mg/10 MJ	7.12	6.78**	7.88***	6.75***	7.88***
Vitamin C	mg/d	110	101	138***	96***	124***	mg/10 MJ	112	108	155***	100**	137***
Sodium	mg/d	4902	4421***	4683*	4497***	4809	mg/10 MJ	5000	4745*	5248*	4695**	5316***
Potassium	mg/d	2880	2403***	2900	2427***	2852	mg/10 MJ	2937	2579***	3250***	2533***	3153***
Calcium	mg/d	573	468***	567	492***	592	mg/10 MJ	585	502***	635**	514***	655***
Magnesium	mg/d	309	268***	294**	275***	294***	mg/10 MJ	315	287***	330**	287***	325*
Phosphorus	mg/d	1242	1079***	1159***	1118***	1203**	mg/10 MJ	1267	1158***	1299	1167***	1330***
Iron	mg/d	9.06	7.22***	8.63*	7.38***	8.66**	mg/10 MJ	9.2	7.7***	9.7*	7.7***	9.6*
Zinc	mg/d	9.75	8.81***	9.10***	9.02***	9.26***	mg/10 MJ	9.9	9.4**	10.2	9.4***	10.2*
Copper	mg/d	1.39	1.23***	1.36	1.24***	1.33**	mg/10 MJ	1.42	1.31***	1.50***	1.29***	1.46*
Manganese	mg/d	4.18	4.26	4.00	4.26	3.93**	mg/10 MJ	4.27	4.58*	4.48*	4.46	4.34

Abbreviations: DR = semi-weighed dietary records; DHQ1 = first self-administered comprehensive diet history questionnaire; BDHQ1 = first brief DHQ; mDHQ = mean of 4 DHQs; mBDHQ = mean of 4 BDHQs.

All variables were log-transformed before analysis. Means were back-transformed.

^a^Energy adjustment was performed according to the residual method and density method.

^b^Mean values obtained by the crude and residual methods were the same. The results of the paired *t*-test for the residual method were shown.

^c^Sum of eicosapentaenoic acid, docosapentaenoic acid, and docosahexaenoic acid.

^d^Sum of retinol, β-carotene/12, α-carotene/24, and cryptoxanthin/24.

^e^Sum of β-carotene, α-carotene/2, and cryptoxanthin/2.

Significant difference from the DR: *P < 0.05, **P < 0.01, ***P < 0.001 (paired *t*-test).

Pearson correlation coefficients for estimates of crude and energy-adjusted nutrient intakes and energy intake on the DR versus the DHQ and BDHQ are shown in Table 4 for women and Table 5 for men. For women, the correlation values of energy with the DR were 0.30 for DHQ1 and 0.29 for BDHQ1; for men, the respective values were 0.41 and 0.23. Additionally, correlation values of energy with the DR for women were 0.38 for mDHQ and 0.42 for mBDHQ; for men, the respective values were 0.49 and 0.38. For nutrients expressed as crude values, median (interquartile range) correlation coefficients for women were 0.40 (0.34 to 0.47) for DHQ1 and 0.39 (0.32 to 0.48) for BDHQ1; for men, the respective values were 0.40 (0.33 to 0.45) and 0.34 (0.25 to 0.40). For energy-adjusted nutrient intakes, almost all correlation values were improved as compared with crude values. Regarding energy-adjusted nutrient intakes by the residual method, median correlation values for women were 0.50 (0.43 to 0.58) for DHQ1 and 0.49 (0.38 to 0.57) for BDHQ1; for men, the respective values were 0.45 (0.37 to 0.55) and 0.51 (0.38 to 0.59). The results of correlation coefficients of energy-adjusted nutrients using the density method were similar to those of the residual method. For energy-adjusted nutrients using the density method, median correlation coefficients for women were 0.49 (0.43 to 0.58) for DHQ1 and 0.49 (0.38 to 0.57) for BDHQ1; for men, the respective values were 0.47 (0.36 to 0.56) and 0.49 (0.36 to 0.59) (data not shown). All deattenuated correlation values were 1.0 to 1.4 times the respective original values. For deattenuated correlations of energy-adjusted nutrient intakes by the residual method, median values for women were 0.57 (0.50 to 0.64) for DHQ1 and 0.54 (0.45 to 0.61) for BDHQ1, with respective values for men of 0.50 (0.42 to 0.59) and 0.56 (0.41 to 0.63). Almost none of the correlation coefficients for DR and BDHQ1 differed from the respective values for DR and DHQ1. Medians of correlation coefficients between the DR and mDHQ or mBDHQ were slightly higher (1.1 to 1.4 times) than the respective values for DHQ1 and BDHQ1. Further, almost none of the correlation coefficients for DR and mBDHQ differed from the respective values for DR and mDHQ.

**Table 4.** Pearson correlation coefficients between 16-day DR and DHQ1, BDHQ1, mDHQ, and mBDHQ for estimates of crude and energy-adjusted^a^ nutrient intakes and energy intake among 92 women

	Crude				Energy-adjusted by the residual method				Energy-adjusted by the residual method and de-attenuated			
		
	DHQ1	BDHQ1	mDHQ	mBDHQ	DHQ1	BDHQ1	mDHQ	mBDHQ	DHQ1	BDHQ1	mDHQ	mBDHQ
Energy	0.30	0.29	0.38	0.42	—	—	—	—	0.32	0.31	0.40	0.45
Protein	0.27	0.26	0.36	0.39	0.47	0.35	0.45	0.44	0.52	0.38	0.50	0.49
Fat	0.44	0.39	0.53	0.49	0.57	0.56	0.64	0.60	0.62	0.61	0.70	0.65
Saturated fat	0.55	0.48	0.64	0.59	0.69	0.64	0.70	0.68	0.75	0.70	0.77	0.74
Monounsaturated fat	0.47	0.44	0.56	0.53	0.52	0.61	0.61	0.63	0.57	0.66	0.66	0.68
Polyunsaturated fat	0.30	0.32	0.37	0.38	0.35	0.41	0.51	0.46	0.42	0.49	0.61	0.55
*n*-6 polyunsaturated fat	0.36	0.38	0.42	0.42	0.42	0.49	0.57	0.53	0.50	0.59	0.68	0.64
*n*-3 polyunsaturated fat	0.19	0.20	0.23	0.32	0.29	0.27	0.40	0.38	0.36	0.34	0.50	0.48
Marine-origin *n-3* polyunsaturated fat^b^	0.35	0.24	0.41	0.37	0.42	0.34	0.53	0.46	0.55	0.43	0.68	0.59
Eicosapentaenoic acid	0.38	0.28	0.45	0.42	0.44	0.40	0.57	0.51	0.58	0.52	0.74	0.67
Docosahexaenoic acid	0.33	0.22	0.38	0.35	0.41	0.30	0.49	0.42	0.53	0.40	0.64	0.56
α-linolenic acid	0.27	0.32	0.31	0.38	0.22	0.36	0.39	0.39	0.27	0.45	0.49	0.49
Cholesterol	0.37	0.32	0.41	0.33	0.31	0.28	0.39	0.31	0.39	0.34	0.47	0.38
Carbohydrate	0.43	0.36	0.48	0.49	0.58	0.48	0.67	0.63	0.62	0.51	0.71	0.67
Total dietary fiber	0.53	0.56	0.58	0.60	0.68	0.65	0.77	0.73	0.71	0.68	0.80	0.76
Soluble dietary fiber	0.50	0.54	0.53	0.57	0.64	0.63	0.72	0.71	0.69	0.67	0.77	0.75
Insoluble dietary fiber	0.53	0.56	0.58	0.60	0.69	0.65	0.76	0.72	0.71	0.68	0.79	0.75
Alcohol	0.84	0.84	0.90	0.84***	0.85	0.84	0.89	0.84*	0.87	0.87	0.91	0.87*
Retinol	0.43	0.42	0.45	0.45	0.43	0.39	0.43	0.42	0.61	0.54	0.60	0.59
Vitamin A (retinol equivalent)^c^	0.45	0.45	0.44	0.48	0.47	0.46	0.42	0.47	0.59	0.57	0.53	0.59
α-carotene	0.49	0.41	0.54	0.51	0.47	0.38	0.50	0.46	0.59	0.48	0.63	0.58
β-carotene	0.54	0.50	0.59	0.55	0.58	0.51	0.63	0.56	0.64	0.57	0.70	0.62
β-carotene equivalent^d^	0.54	0.48	0.60	0.57	0.58	0.51	0.66	0.60	0.64	0.56	0.73	0.66
Cryptoxanthin	0.42	0.34	0.54	0.48	0.48	0.38	0.61	0.51	0.58	0.46	0.73	0.61**
α-tocopherol	0.30	0.31	0.38	0.42	0.41	0.42	0.54	0.56	0.47	0.48	0.62	0.65
Vitamin K	0.41	0.50	0.46	0.50	0.46	0.58	0.61	0.63	0.50	0.64*	0.67	0.69
Thiamin	0.34	0.33	0.45	0.48	0.36	0.35	0.35	0.49	0.46	0.45	0.44	0.62*
Riboflavin	0.37	0.35	0.43	0.49	0.53	0.52	0.50	0.64*	0.57	0.56	0.54	0.69**
Niacin	0.38	0.35	0.46	0.48	0.52	0.37*	0.48	0.44	0.57	0.40*	0.53	0.49
Vitamin B_6_	0.47	0.38	0.51	0.47	0.63	0.49*	0.67	0.57	0.68	0.52*	0.71	0.61
Vitamin B_12_	0.38	0.28	0.34	0.34	0.47	0.31	0.43	0.40	0.58	0.39*	0.53	0.50
Folate	0.46	0.52	0.52	0.58	0.52	0.59	0.61	0.65	0.55	0.62	0.64	0.68
Pantothenic acid	0.34	0.33	0.44	0.45	0.60	0.54	0.67	0.64	0.65	0.59	0.73	0.70
Vitamin C	0.45	0.55	0.55	0.63	0.49	0.63	0.64	0.70	0.52	0.66*	0.68	0.74
Sodium	0.31	0.39	0.40	0.51	0.35	0.44	0.43	0.55	0.39	0.49	0.48	0.61
Potassium	0.33	0.42	0.45	0.52	0.50	0.56	0.61	0.66	0.53	0.59	0.64	0.68
Calcium	0.36	0.36	0.49	0.48	0.53	0.51	0.64	0.63	0.56	0.54	0.68	0.67
Magnesium	0.39	0.42	0.46	0.48	0.61	0.56	0.67	0.65	0.64	0.59	0.70	0.69
Phosphorus	0.28	0.29	0.41	0.42	0.48	0.38	0.53	0.53	0.51	0.41	0.56	0.56
Iron	0.44	0.45	0.46	0.50	0.63	0.53	0.64	0.62	0.67	0.57	0.69	0.66
Zinc	0.35	0.29	0.43	0.37	0.50	0.32*	0.59	0.38**	0.60	0.39**	0.71	0.45***
Copper	0.49	0.50	0.52	0.54	0.62	0.57	0.68	0.66	0.66	0.61	0.73	0.71
Manganese	0.48	0.58	0.67	0.70	0.47	0.67*	0.67	0.75	0.48	0.69**	0.69	0.77

Abbreviations: DR = semi-weighed dietary records; DHQ1 = first self-administered comprehensive diet history questionnaire; BDHQ1 = first brief DHQ; mDHQ = mean of 4 DHQs; mBDHQ = mean of 4 BDHQs.

All variables were log-transformed before analyses.

^a^Energy-adjustment was performed according to the residual method and density method. The results using the density method are not shown.

^b^Sum of eicosapentaenoic acid, docosapentaenoic acid, and docosahexaenoic acid.

^c^Sum of retinol, β-carotene/12, α-carotene/24, and cryptoxanthin/24.

^d^Sum of β-carotene, and α-carotene/2, and cryptoxanthin/2.

Significant difference between correlation coefficients of the DHQ and BDHQ: *P < 0.05, **P < 0.01, ***P < 0.001 (Meng-Rosenthal-Rubin method).

**Table 5.** Pearson correlation coefficients between 16-day DR and DHQ1, BDHQ1, mDHQ, and mBDHQ for estimates of crude and energy-adjusted^a^ nutrient intakes and energy intake among 92 men

	Crude				Energy-adjusted by the residual method				Energy-adjusted by the residual method and de-attenuated			
		
	DHQ1	BDHQ1	mDHQ	mBDHQ	DHQ1	BDHQ1	mDHQ	mBDHQ	DHQ1	BDHQ1	mDHQ	mBDHQ
Energy	0.41	0.23	0.49	0.38	—	—	—	—	0.42	0.24*	0.51	0.40
Protein	0.33	0.24	0.43	0.44	0.28	0.38	0.47	0.57	0.31	0.41	0.51	0.62
Fat	0.41	0.37	0.50	0.46	0.52	0.59	0.63	0.65	0.57	0.64	0.69	0.70
Saturated fat	0.50	0.45	0.63	0.54	0.55	0.58	0.67	0.61	0.60	0.63	0.73	0.67
Monounsaturated fat	0.45	0.39	0.51	0.47	0.58	0.61	0.66	0.66	0.63	0.66	0.71	0.72
Polyunsaturated fat	0.33	0.28	0.34	0.38	0.45	0.48	0.47	0.54	0.52	0.56	0.54	0.63
*n*-6 polyunsaturated fat	0.36	0.37	0.39	0.44	0.47	0.55	0.52	0.57	0.54	0.63	0.59	0.65
*n*-3 polyunsaturated fat	0.22	0.04	0.19	0.24	0.33	0.19	0.29	0.41	0.42	0.25	0.38	0.53
Marine-origin *n-3* polyunsaturated fat^b^	0.32	0.20	0.38	0.37	0.38	0.33	0.47	0.51	0.48	0.41	0.59	0.63
Eicosapentaenoic acid	0.32	0.19	0.38	0.36	0.39	0.31	0.47	0.50	0.49	0.39	0.59	0.63
Docosahexaenoic acid	0.33	0.17	0.37	0.37	0.39	0.30	0.45	0.49	0.49	0.38	0.57	0.63
α-linolenic acid	0.25	0.24	0.24	0.30	0.39	0.42	0.37	0.47	0.47	0.51	0.44	0.56
Cholesterol	0.41	0.33	0.51	0.44	0.44	0.33	0.58	0.43*	0.51	0.39	0.68	0.50**
Carbohydrate	0.55	0.35**	0.62	0.51*	0.67	0.64	0.77	0.74	0.70	0.68	0.81	0.78
Total dietary fiber	0.57	0.51	0.65	0.58	0.71	0.69	0.79	0.76	0.73	0.72	0.82	0.79
Soluble dietary fiber	0.53	0.45	0.63	0.54	0.64	0.62	0.76	0.72	0.67	0.65	0.80	0.75
Insoluble dietary fiber	0.55	0.48	0.63	0.55	0.71	0.68	0.78	0.74	0.73	0.71	0.80	0.76
Alcohol	0.81	0.81	0.90	0.89	0.82	0.81	0.90	0.90	0.83	0.83	0.92	0.92
Retinol	0.21	0.10	0.32	0.37	0.20	0.19	0.32	0.38	0.25	0.24	0.40	0.48
Vitamin A (retinol equivalent)^c^	0.23	0.15	0.34	0.41	0.20	0.25	0.34	0.45	0.23	0.30	0.41	0.53*
α-carotene	0.13	0.33*	0.32	0.43	0.08	0.31*	0.28	0.44*	0.09	0.37**	0.33	0.52**
β-carotene	0.36	0.39	0.53	0.52	0.36	0.42	0.58	0.58	0.40	0.47	0.65	0.65
β-carotene equivalent^d^	0.38	0.38	0.53	0.53	0.38	0.42	0.58	0.59	0.42	0.47	0.64	0.66
Cryptoxanthin	0.54	0.34*	0.53	0.53	0.53	0.37*	0.54	0.54	0.61	0.42*	0.62	0.62
α-tocopherol	0.29	0.20	0.33	0.37	0.43	0.47	0.46	0.59	0.49	0.54	0.53	0.67*
Vitamin K	0.41	0.44	0.53	0.59	0.46	0.54	0.64	0.68	0.50	0.59	0.71	0.75
Thiamin	0.31	0.20	0.42	0.40	0.32	0.29	0.35	0.45	0.41	0.37	0.45	0.57
Riboflavin	0.39	0.34	0.54	0.54	0.39	0.56*	0.57	0.71*	0.43	0.61*	0.62	0.77*
Niacin	0.41	0.19*	0.53	0.44	0.40	0.23	0.50	0.46	0.43	0.25	0.55	0.50
Vitamin B_6_	0.41	0.28	0.51	0.45	0.53	0.48	0.59	0.57	0.57	0.51	0.63	0.61
Vitamin B_12_	0.35	0.30	0.38	0.41	0.37	0.40	0.44	0.52	0.42	0.46	0.51	0.60
Folate	0.37	0.38	0.46	0.53	0.35	0.54*	0.50	0.62	0.37	0.58*	0.53	0.66*
Pantothenic acid	0.41	0.35	0.55	0.53	0.56	0.65	0.71	0.76	0.61	0.70	0.77	0.83
Vitamin C	0.44	0.46	0.51	0.62	0.50	0.61	0.57	0.74**	0.52	0.64	0.60	0.78**
Sodium	0.43	0.43	0.47	0.51	0.29	0.51*	0.39	0.54	0.32	0.57*	0.44	0.60*
Potassium	0.42	0.38	0.54	0.52	0.51	0.64	0.67	0.75	0.53	0.66*	0.70	0.77
Calcium	0.54	0.51	0.68	0.60	0.66	0.66	0.77	0.74	0.69	0.68	0.81	0.77
Magnesium	0.40	0.33	0.51	0.44	0.55	0.59	0.64	0.68	0.58	0.62	0.67	0.71
Phosphorus	0.36	0.30	0.50	0.46	0.51	0.52	0.62	0.66	0.53	0.55	0.66	0.70
Iron	0.37	0.40	0.46	0.50	0.45	0.57	0.60	0.69	0.48	0.61	0.64	0.74*
Zinc	0.37	0.33	0.46	0.45	0.31	0.43	0.54	0.56	0.36	0.48	0.61	0.64
Copper	0.47	0.35	0.53	0.48	0.57	0.55	0.67	0.67	0.61	0.59	0.71	0.71
Manganese	0.50	0.43	0.60	0.62	0.49	0.57	0.62	0.68	0.50	0.59	0.64	0.71

Abbreviations: DR = semi-weighed dietary records; DHQ1 = first self-administered comprehensive diet history questionnaire; BDHQ1 = first brief DHQ; mDHQ = mean of 4 DHQs; mBDHQ = mean of 4 BDHQs.

All variables were log-transformed before analyses.

^a^Energy-adjustment was performed according to the residual method and density method. The results using the density method are not shown.

^b^Sum of eicosapentaenoic acid, docosapentaenoic acid, and docosahexaenoic acid.

^c^Sum of retinol, β-carotene/12, α-carotene/24, and cryptoxanthin/24.

^d^Sum of β-carotene, α-carotene/2, and cryptoxanthin/2.

Significant difference between correlation coefficients of the DHQ and BDHQ: *P < 0.05, **P < 0.01 (Meng-Rosenthal-Rubin method).

## DISCUSSION

We examined the relative validity of energy and nutrient intakes estimated by the DHQ and BDHQ, using a 16-day DR as reference. For energy and many (ie, 50%–90%) energy-adjusted nutrients, mean intakes estimated by DHQ1 and BDHQ1 were significantly different from those estimated by the DR. The results for mDHQ and mBDHQ were similar. These results suggest that the DHQ and BDHQ are satisfactory for estimating mean values for only a limited number of nutrients.

However, for many (ie, 57%–83%) correlations with energy-adjustment and many (ie, 79%–90%) deattenuated correlations with energy-adjustment, values greater than 0.4 were observed between the DR and DHQ1 and between the DR and BDHQ1. Although the correlation with the DR was slightly higher for mDHQ and mBDHQ than for DHQ1 and BDHQ1, the latter questionnaires had reasonable correlations for many nutrient intakes. Furthermore, correlation coefficients between the DR and DHQ1 and between the DR and BDHQ1 did not significantly differ for almost all nutrients, which was also the case for mDHQ and mBDHQ. Thus, the DHQ and BDHQ had satisfactory ranking ability for many nutrients in this population.

Of the several US studies that have compared short and long versions of the same questionnaire with regard to energy and nutrient intake,^5^^–^^7^ all have shown reasonable correlations between the 2 versions. Our results in a Japanese population are consistent with those findings. Wakai reviewed the validity of dietary questionnaires developed and validated in Japan^16^ and showed that medians of coefficients between DRs and questionnaires on energy and nutrients ranged from 0.31 to 0.56 in the studies investigated. Our present DHQ and BDHQ thus performed similarly to these other Japanese dietary questionnaires. Wakai then considered long (97 or more food items) versus short questionnaires (<70 items) and reported that the median correlation coefficient for nutrients in an individual questionnaire ranged from 0.42 to 0.52 for the long form and from 0.31 to 0.45 for the short form. On the basis of those results, he concluded that long questionnaires had slightly higher validity in the estimation of nutrient intake. In the present study, the corresponding values for women were 0.57 for the DHQ and 0.54 for the BDHQ, with respective values for men of 0.50 and 0.56. Our study indicated that, unlike the results of the review, the BDHQ did not necessarily have a worse correlation as compared with the DHQ.

There are several explanations for why both the 150-item DHQ and the 58-item BDHQ had relatively high correlations. First, the food and beverage items in the BDHQ may accurately reflect the foods commonly consumed in Japan. Second, the DHQ requires more time to complete than the BDHQ, which may reduce the accuracy of responses for the former questionnaire. Third, the BDHQ differs from the DHQ in that it does not ask about portion size. Given that misestimation of portion size is a source of error in answering these questionnaires, some participants might have inaccurately answered at least some of the DHQ questions on portion size.^17^ Although it is not clear why the median correlation coefficient of the DHQ was higher than that of the BDHQ in women while the opposite was true in men, the degree of the effects of questionnaire length and misestimation of portion size may differ by sex. Because of its lesser burden, the BDHQ might be preferred in the investigation of many of the nutrients examined here. However, it should be mentioned that correlations of some nutrients (eg, zinc in women and cryptoxanthin in men) were higher for the DHQ than for the BDHQ. Further, the DHQ was better than the BDHQ in estimating mean intakes of vitamin C and some other nutrients. These results indicate that the DHQ is more suitable than the BDHQ in estimating intakes of some nutrients.

Although the median correlations for both the DHQ and BDHQ were relatively good, in men, correlation coefficients for retinol in the DHQ1 and BDHQ1, α-carotene in the DHQ1, and n-3 polyunsaturated fatty acid in the BDHQ1 were much lower than those of other nutrients in the present study and much lower than those of any nutrient in other studies.^18^^–^^24^ However, the correlations of these nutrients from mDHQ and mBDHQ were slightly higher than those from DHQ1 and BDHQ1. If we were to examine the intakes of these nutrients in an epidemiologic study using the DHQ or BDHQ, multiple DHQs and BDHQs would be needed.

Several limitations of this study should be mentioned. First, although we assumed that energy and nutrient intakes derived from the DR were the standard, DRs are susceptible to measurement error due to erroneous recording and potential changes in eating behavior. Nevertheless, as compared with 24-hour dietary recall or other instruments that rely on memory, errors in DRs are thought to have a lower correlation with errors in the DHQ and BDHQ.^1^ Biomarkers are a better standard for some nutrients, and any errors in such markers are independent of errors in the questionnaire. Validity testing of the DHQ for Japanese adults by using 24-hour urine, serum, or doubly labeled water methods has revealed satisfactory validity for some nutrients.^9^^–^^11^ Validation studies with quantitative biomarkers are also necessary for the BDHQ. Second, because the algorithm used to calculate food intake from the BDHQ was written using previous information^19^ and other unpublished observations, it had insufficient reliability. Finally, the generalizability of the present results is limited because the participants were not representative of the general Japanese population and might have been highly health conscious. Additionally, while all the women answered the questionnaires themselves, a considerable number of men (32%) answered with the help of their wives. The validity of the 2 questionnaires in men might have been lower had they answered the questionnaires themselves.

In conclusion, this study showed that the DHQ and BDHQ had satisfactory ranking ability for energy-adjusted intakes of many nutrients, as compared with DRs, in a population of Japanese men and women, although ability of these instruments to estimate mean values was satisfactory for only a limited number of nutrients. Additionally, although the correlations obtained from mDHQ and mBDHQ were better than the respective values from DHQ1 and BDHQ1, even a single DHQ and BDHQ had reasonable correlations for many nutrient intakes. The validity of the BDHQ was similar to that of the DHQ. These findings support the idea that, for many energy-adjusted nutrients, both the DHQ and BDHQ can be used in large-scale epidemiologic studies in Japan.
